# Can Space Tourism Boost Sustainable Behavior?

**DOI:** 10.3389/fpsyg.2021.771936

**Published:** 2021-11-19

**Authors:** Nicola Mammarella

**Affiliations:** Department of Psychological Sciences, Health and Territory, University of Chieti, Chieti, Italy

**Keywords:** space science, sustainable behavior, positive emotions, environment, emotion

## Introduction

As the golden age of space tourism is becoming a reality, many are wondering about the usefulness of sending people to space for a few minutes. Given the high cost of a spaceflight, pollution-related problems, and the rigid training that private astronauts undergo, it is fundamental to understand and predict whether people can derive benefits from this type of tourism also from a psychological and behavioral point of view. Here we ask whether participating in a spaceflight may shape human behavior in terms of sustainable behavior (e.g., prosocial skills). The question may be perceived as a non-sense. In fact, spatial industries and rockets are far from being an example of sustainability. However, the definition of sustainable behavior appears to offer a different perspective that may add to the psychological value of space tourism per sé at least from a theoretical point of view.

### Sustainable Behavior and Positive Emotions

Sustainable behavior can be defined as a series of voluntary actions that result in benefits for the natural environment and for the whole humanity. Prosocial behaviors are an instance of sustainable actions when referring to helping people and doing something for the conservation of their natural environment (e.g., Eisenberg, 1982). Sustainable behaviors are based on the assumption that it is important to understand the complexity of the natural environment and to become aware of the consequences resulting from our behaviors as they impact over Earth's integrity.

One of the main characteristics of sustainable behavior is that sustainability and positive emotions are linked conceptually and empirically. In fact, positive emotions are thought to foster subsequent helping behavior and vice versa. For example, there are studies showing that inducing the idea of love by asking to retrieve memory of a love episode, had a significant positive effect on compliance to a request by a passerby who was asked for help (e.g., Lamy et al., 2008). When measured via self-reports of intent to help or experimental records of helping behaviors, individuals that engage in sustainable actions frequently report greater level of satisfaction, self-efficacy and, generally speaking, psychological well-being (e.g., Fredrickson and Joiner, 2018).

As theorized by Barbara Fredrickson and her colleagues (e.g., Fredrickson and Joiner, 2018), the main idea is that positive emotions are associated with greater feelings of self–other overlap. That is, by broadening cognition, positive emotions produce more inclusive social categorization and subsequently produce feelings of oneness, helping behavior toward people and their natural environment.

In this regard, one can wonder whether space tourism may offer a contribution.

For instance, Jeff Bezos' first words after returning to Earth “*I felt unbelievably good …this is the only good planet in the solar system, and we have to take care of it. When you go to space and see how fragile it is you want to take care of it even mor*e” seem to testify how positive emotions fuel people's desire to experience conditions of helping behavior and engaging in conservation practices.

However, one can argue that the association between positive emotions and sustainable behavior is not always straightforward and this may be particularly true when referring to an extreme environment such as space. In fact, a space mission is characterized by many physical and psychological stressors such as microgravity, isolation, confinement, sensory and sleep deprivation (e.g., Messerotti Benvenuti et al., 2011, 2013; Spironelli and Angrilli, 2011) that may differentially impact on affective responses and on the subsequent engagement in sustainable actions.

Moreover, a study by Ballantyne et al. (2008) found that a visit to a botanic garden, thought to foster positive emotions (e.g., enjoying being in a nature scene, admiring a garden's scenery with family, etc.) coupled with the importance of preserving plants, did not generate a higher level of interest in and commitment to conservation practices compared with other types of visits (e.g., museum, zoo, etc.). This finding indicates that perceiving vulnerability does not necessarily lead to the subsequent adoption of sustainable behaviors.

The rationale being that sustainable actions do not simply rely on the sole exposure to a certain environment, rather it requires the interaction of multiple processes, such as decision making, emotion, motivation, attention, etc. (to cite only few) that can lead individuals to act in different ways. The same scenario, thus, may occur during a space mission: positive emotions may be associated to space tourism but not necessarily in a way that fosters behavioral changes.

### The “Perception of the Earth” Effect

One aspect that may strengthen the link between space tourism and sustainable behavior is the focus on Earth's perception as thought to be strictly connected with the occurrence of positive emotions and with subsequent sustainable behaviors (e.g., Suedfeld et al., 2010).

Basically, the global vision of the Earth prompts the occurrence of positive emotions. This phenomenon looks like a mood induction procedure where participants are exposed to a series of positively laden pictures or to a funny movie with the aim of increasing their mood on the positive side. The rationale being that when measuring the degree to which people see the “big picture” or focus on smaller details and its relationship with positive emotions, it was found that compared with those in negative or neutral states, people who experience positive emotions tend to focus on global processing (e.g., Fredrickson and Branigan, 2005).

This finding may indirectly indicate that perceiving the Earth may alone trigger positive emotions or, to say it better, that global visual processing and the generation of positive emotions are somehow linked. For example, in his diary, cosmonaut Lebedev wrote that the vision of Earth was restful and positive to him and helped him to cope with his 211-day *Salyut 7* mission (e.g., Kanas and Manzey, 2008). In addition, the type and the number of Earth pictures taken daily by astronauts during their mission on the International Space Station communicate the need of acting for conservation of Earth's beauty.

By studying reports of retired astronauts, researchers concluded that being in space is indeed reported as a massive experience with a long-lasting impact on their psychological well-being (e.g., Kanas and Manzey, 2008). In particular, their experience has been described with the following categories: perception of the Earth, perception of Space, new possibilities, appreciating life, personal strength, changes in daily life, relating to others, spiritual change (these categories were also adopted by the so-called Positive Effects of Being in Space or PEBS which assesses the positive attitude toward space). In another study positive changes were measured and compared between a sample of 20 retired male Mir and International Space Station cosmonauts and two groups on Earth who had experienced stressful events (e.g., Suedfeld et al., 2010). Cosmonauts' scores resulted particularly high in the field of realization of new possibilities and personal strength. Moreover, those who had spent more than a year in space and those who had flown to both Mir and the ISS were more likely to report a positive change in their appreciation of the others and in their willingness to act to preserve our planet. Critical here are gender differences. In fact, the “tend-and-befriend” strategy (e.g., Taylor et al., 2000) mostly used by female compared with male astronauts during space mission has been shown to be oriented toward promoting prosocial behavior to a greater extent (e.g., team cohesion and team care). Indeed, while high competitiveness and poor sharing of personal concerns usually characterize all-male expedition teams, women tend to worry about the crewmates well-being and the decrease in crew cohesion more (e.g., Kanas and Manzey, 2008).

It is important to notice that the above-mentioned data come from studies where professional astronauts were involved. Indeed, there are differences among professional astronauts and private astronauts, for instance, in terms of long duration training, motivation, skills, education etc. Consequently, the relationship between Earth perception, positive emotions and sustainable behavior should be taken with caution when referring to private space tourism. Due to the lack of data, we can only assume that the association between Earth perception and positive emotions may be similar to the one reported by professional astronauts (e.g., Kanas and Manzey, 2008; Alfano et al., 2018), for example, in terms of a more positive view of themselves and the others and of a better sense of the unity of humankind (e.g., Kanas and Manzey, 2008). Reports from private astronauts seem to support our assumption, but we need future studies to help unraveling this interaction better.

## Discussion

In closing, we emphasize that the findings reported here coming from behavioral studies on Earth and during space missions with professional astronauts seems to support the idea that space tourism too can generate positive emotions and, in turns, facilitate sustainable and approach behaviors. Of course, motivating people to engage in helping behaviors (e.g., interpersonal trust, providing social support, conservation practices) cannot rely on the sole perception of Earth's fragility. However, space tourism may represent a challenging and interesting new avenue for future research in this domain. Nevertheless, we hope that space tourism can offer the benefits of leading to more sustainable actions, capable of responding to the needs of most rather than those of few. In this way, space tourism may offer unexpected opportunities in terms of action for conservation strategies and aid programs also from a psychological point of view.

## Author Contributions

NM developed the concept of the manuscript and the drafting.

## Funding

This study was funded by the Department of Psychological Sciences, Health and Territory, University of Chieti.

## Conflict of Interest

The author declares that the research was conducted in the absence of any commercial or financial relationships that could be construed as a potential conflict of interest.

## Publisher's Note

All claims expressed in this article are solely those of the authors and do not necessarily represent those of their affiliated organizations, or those of the publisher, the editors and the reviewers. Any product that may be evaluated in this article, or claim that may be made by its manufacturer, is not guaranteed or endorsed by the publisher.
